# Bovine herpes virus-1 (BoHV-1) in cattle–a review with emphasis on reproductive impacts and the emergence of infection in Ireland and the United Kingdom

**DOI:** 10.1186/2046-0481-66-15

**Published:** 2013-08-01

**Authors:** David A Graham

**Affiliations:** 1 Animal Health Ireland, Main Street, Carrick on Shannon, County Leitrim, Ireland

**Keywords:** BoHV-1, IBR, Reproduction, Fertility, Abortion, Ireland, United Kingdom

## Abstract

Bovine reproductive disease attributable to bovine herpes virus-1 (BoHV-1) was first described in Germany in the 19^th^ century, being recognised primarily as the cause of infectious vulvovaginitis and balanoposthitis until the mid-1950s when a more virulent strain of the virus (BoHV-1.1) associated with respiratory disease (infectious bovine rhinotracheitis; IBR) emerged in the western United States. Subsequently, IBR emerged as a clinical condition in Europe, from the 1970s onward. While the ability of BoHV-1 to produce respiratory disease is now well recognised, the potential negative outcomes of infection on fertility and reproduction are less frequently considered. This review was conducted against the background of the prioritization of disease caused by BoHV-1 as one of several diseases to be addressed by Animal Health Ireland, with the twin goals of summarizing the published literature on the potential outcomes of infection at different stages of breeding and pregnancy, and of describing the emergence of BoHV-1 as a significant pathogen in Ireland and the UK.

## Introduction

Addressing infection with bovine herpes virus-1 (BoHV-1) in the Irish cattle population has been identified as a priority for Animal Health Ireland (AHI; http://www.animalhealthireland.ie), a not-for profit partnership between livestock farmers, processors, service providers and government to address important non-regulated diseases that was established in 2009 [1,2]. More recently, Animal Health and Welfare NI (AHWNI; http://www.animalhealthni.com) has been established in Northern Ireland (part of the United Kingdom) with a similar remit, offering the possibility of dealing with BoHV-1 and other prioritized non-regulated diseases on an all-island basis.

As will be described later in detail, the respiratory form of BoHV-1 infection spread to Europe in the late 1960s and early 1970s and since then infectious bovine rhinotracheitis (IBR) has been considered to be the predominant clinical disease associated with this virus, including in Ireland. As a consequence, there has been relatively little study or recognition of the reproductive impact of BoHV-1 infection in recent years. In order to better inform the debates being led by AHI and AHWNI in the Republic of Ireland and Northern Ireland, this review was conducted with two primary goals. Firstly, drawing on published case reports, experimental trials and epidemiological studies to highlight the negative outcomes on reproductive performance that may be associated with BoHV-1. These may be divided into the impacts on conception and the early stages of pregnancy (fertility) and the ability of the virus to induce abortion in mid to late gestation. The second goal was to review the emergence of BoHV-1 in Ireland and the United Kingdom (UK) as a cause of clinical disease and describe the hypothesis put forward to explain this.

## Review

### BoHV-1: historical perspective

The first report of disease believed to be due to bovine herpesvirus-1 (BoHV-1) came from Germany in the 19^th^ century where a condition referred to as Bläschenausschlag, or coital vesicular exanthema (CVE) was described. This venereal disease was subsequently shown in 1928 to have a viral aetiology when it was demonstrated that it could be transmitted by a filterable agent [3]. Thereafter CVE, more commonly referred to as infectious pustular vulvo-vaginitis in cows and heifers (IPV) and infectious pustular balanoposthitis in bulls (IPB) remained the primary recognised manifestation of infection with BoHV-1 until the 1950s.

In 1954 a new clinical condition affecting dairy herds in the Los Angeles area of California was described [4]. Following the index case in October 1953, 51 further herds in the same area became affected over the next 4 months. Typical presenting signs included a high fever (38.9–42.2°C) and a sudden drop in milk yield which in many cases ceased completely within 24–48 hours. Excessive salivation was commonly reported and the mucous membranes of the nares were inflamed with a nasal discharge which was initially mucus and later mucopurulent. A short explosive cough was also characteristic of the disease. Morbidity in these herds was 7.6% with a mortality rate among infected animals of 3%. Affected animals returned to normal fairly rapidly with evidence of the acute phase typically disappearing within one month. On post-mortem examination the most prominent and characteristic lesion was severe haemorrhagic tracheitis and bronchitis. While diarrhoea was not a clinical sign, severe enteritis of the small intestines and mucoid enteritis of the large intestines were reported. The aetiology was believed to be viral and this report is now considered to be the first peer-reviewed description of infectious bovine rhinotracheitis (IBR) in cattle.

The following year (1955), a report was published [5] describing an emerging respiratory disease of feedlot and dairy cattle which it named infectious necrotic rhinotracheitis. This report was the first to use the term “red nose” in relation to this disease, in reference to the marked inflammation of the nasal pad and passages. This was first observed in the autumn of 1950 in Colorado and consistent with the report from the previous year [4], was characterised by a high fever and acute inflammation affecting the upper respiratory tract down to and including the bronchi. Following its emergence in Colorado in 1950, signs were observed in a number of feedlots between 1951 and 1953 with affected cattle to this point typically being mature animals. This changed in 1954 when the disease was recognised in calves as young as 3 weeks of age and also occurred in epizootic form in dairy herds. Clinical signs were again similar to those described in the index case [4] with a sharp reduction in milk yield including cessation in some animals with recovery in 5–7 days and changes in the gastro-intestinal tract with mucosal ulceration in the abomasum, severe enteritis in the small intestine and mild inflammation in the large intestine. Unlike the index case diarrhoea was reported as a presenting sign in some cases, with animals having fresh or digested blood in their dung which in some instances was linked to abomasal ulceration.

Based on the results of restriction endonuclease analysis, isolates of BoHV-1 can be subdivided into three subtypes. BoHV-1 type 1 (BoHV-1.1) isolates are typically associated with respiratory disease and abortion and are sometimes referred to as being IBR virus-like. A second group of viruses, referred to as BoHV-1.2 are typically associated with venereal infection and are referred to as being IPV-like. This grouping can be further subdivided into BoHV-1.2a and −1.2b, being clinically distinguished by the association of the former with abortions, although these distinctions between subtypes are not absolute [3,6-8].

### Fertility

#### Experimental studies at service/oestrus

The potential for BoHV-1 infection to have a negative impact on fertility has been recognised for many years. One of the earliest reports, published in 1967 described an experimental study to investigate the impact of the presence of BoHV-1 in semen used in artificial insemination (AI), [9]. Initially, 4 heifers were inseminated with semen spiked with the U.S. BoHV-1 strain K22 (BoHV-1.2b, IPV-like; [10]), with the semen being deposited in the body of the uterus. Each heifer showed clinical signs of IPV, with 3 returning to oestrus 9–13 days later while the fourth conceived and ultimately produced a live calf at term. In a follow-up study 6 of 8 seronegative heifers inseminated in the same way had short oestrus cycles (11–15 days). Endometrial biopsies indicated the development of a chronic necrotising endometritis which was still evident 31–47 days after insemination. Histopathological changes were also observed in the vulva, vagina and oviducts of some animals at this stage and 5 of 8 animals examined had cystic corpora lutea.

A subsequent more detailed study, [11] investigated the effect of BoHV-1 on breeding by both AI and natural service. Seronegative heifers and cows were either inseminated with semen and an Australian strain of BoHV-1 or were mated naturally to bulls which had been inoculated preputially 2 days previously with the same virus. Following AI, when semen was deposited into the uterus, only 2 of 10 animals conceived to first service, with a further 2 animals conceiving to a second BoHV-1-free insemination. In addition 4 of the 6 non-pregnant animals showed one or more shortened oestrus cycles. Overall there were 4.5 services per conception in this group as compared to 1.7 services per conception in a control group where 9 of 10 conceived within 2 inseminations with no incidences of shortened oestrus cycles. In contrast, breeding by natural service produced similar outcomes independent of infection status. All 10 animals bred by non-infected bulls were pregnant after 2 services whereas 9 of 10 bred by BoHV-1-infected bulls were in calf with respective services per conception of 1.2 and 1.4.

Irrespective of the means of service all animals receiving BoHV-1-infected semen developed lesions of IPV within 48 hours which regressed 9–11 days later, with the inoculated bulls developing IPB. In the artificially inseminated group, virus was recovered consistently from vaginal swabs from all animals with a mean duration of excretion of 8.8 days. Virus was intermittently recovered from some animals for as long as 40 days post insemination. Histopathological examination of the 6 non-pregnant animals in the AI group showed lesions of chronic endometritis ranging in nature from mild to severe. Lesions were also recorded in the oviduct, vagina and Bartolin’s glands.

The authors concluded that the route of infection is critical in determining whether endometritis and infertility occur. While the introduction of BoHV-1 by natural service does not appear to significantly affect fertility, the inoculation of BoHV-1 into the uterus can cause infertility due to endometritis and also increase the incidence of shortened oestrus cycles.

The potential negative impact of the use of BoHV-1-contaminated semen was also highlighted by a field study which who reported that cows in 20 herds inseminated with contaminated semen had a non-return rate of 13.4% compared with 60.8% when semen from other bulls was used [12]. In addition 22.1% of cows returning to service in the infertile group had shortened oestrus cycles.

In the 1980’s an American research group highlighted the lack of information concerning the effect of BoHV-1 on the reproductive organs of cattle that were non-pregnant or in early gestation [13] and conducted a number of studies to investigate this. In the first of these [13], 12 seronegative heifers were inoculated by the intrauterine route one day after natural mating with a non-infected bull. 4 heifers received strain FI (Foetal Iowa) (type BoHV-1.2a [8]) while the others received one of two other strains–(Iowa or Colorado; type BoHV-1.1 [8]). Animals were subject to post mortem examination 4–14 days later. The corpus luteum (CL) of 8 of the 12 virus-inoculated heifers, representing all 3 inocula, contained cysts. No gross or microscopic lesions were observed in the control heifers or those inoculated with the FI isolate. In contrast, gross lesions of oedema, haemorrhage and necrosis were recorded in the uterine bodies or horns of the heifers that were inoculated with either of the other strains. Microscopic lesions ranging from mild focal endometritis to severe diffuse necrotizing metritis were also observed in these groups. In addition to the luteal inflammation, 2 heifers had areas of necrosis in the contra-lateral ovary, with one having a severe, diffuse, necrotizing oophoritis.

Based on these findings, the authors drew a number of conclusions. Firstly, the absence of gross lesions in the cranial uterine horns, accompanied by minimal microscopic changes, were unlikely to interfere with blastocyst attachment in this area 3 weeks after conception and therefore the infertility associated with intra-uterine exposure is unlikely to be a direct consequence of the pathological effects of the virus on the uterine epithelium.

Secondly, BoHV-1 can produce inflammation and necrosis of the CL after intra-uterine exposure at oestrus. However, they concluded that the intra-luteal cysts are probably not a direct result of viral damage due to the lack of correlation between their presence and the detection of lesions or the isolation of virus (which was not isolated from all corpora lutea) in challenged cattle and their occurrence in some control animals also.

The difference in outcomes between BoHV-1 isolates is noteworthy. In a follow up study [14] the Colorado and Iowa isolates were used to infect seronegative heifers on the day after breeding by natural service. For each virus isolate, 2 heifers were each inoculated by the intravenous (i.v.), intramuscular (i.m.) and intra-nasal (i.n.) routes, with animals being subject to post mortem examination 11–15 days later. Gross ovarian lesions were found in the ovaries of 4 heifers (3 i.v., 1 i.m.). Microscopic ovarian lesions were found in all 4 animals inoculated by the i.v. route and 3 of 4 by the i.m. route, with virus being recovered from the ovaries of each of these animals but not from other parts of the reproductive tract. No gross or microscopic lesions were detected in the ovaries of animals exposed by aerosol route and virus was not isolated from their ovarian tissue. The authors concluded that BoHV-1 readily gains access to ovarian tissue from the blood and that the immediate post-oestrus ovary is particularly susceptible to infection via this route. They proposed that the absence of ovarian lesions following aerosol infection probably reflects the absence of a viraemia with the incidence of ovarian involvement related to the duration and height of post-infection viraemia.

The Iowa and Colorado isolates were then used to conduct a further study [15] to investigate the effect of BoHV-1-induced oophoritis on ovarian function during an acute primary i.v. infection and after recovery and reactivation of latent infection. Six heifers were inoculated by the i.v. route with either Iowa (n = 3) or Colorado (n = 3) isolates at oestrus following natural service. Plasma progesterone was followed over subsequent cycles to monitor CL function and was found to be depressed in all heifers. The reduction in progesterone was more marked with the Iowa isolate. Two heifers had a normal next oestrus cycle whereas for 3, onset was delayed by a few days to a week and the one remaining heifer returned to normal cyclicity after 2 months. The authors concluded that while primary BoHV-1 infection at oestrus may cause severe oophoritis, the resulting functional impairment of the ovary is temporary. None of the heifers became pregnant, although the authors were unable to state whether this reproductive failure was as a result of necrosis of the CL or lethal embryonic infection. Despite the i.v. route of administration, virus was isolated from the nasal (n = 5), vaginal (n = 6) and rectal (n = 1) mucosal surfaces from the six heifers after reactivation. No lesions were observed on the ovaries of any heifer and virus was only isolated from the ovarian tissue of one animal, suggesting that recurrent BoHV-1 infection does not directly interfere with ovulation or luteal function.

To compare the pathological changes induced in the ovaries by different modified live IBR vaccines administered during oestrus, 22 seronegative heifers were synchronised with 2 doses of prostaglandin and then administered double doses by the intravenous route of one of three modified live vaccines licenced in North America [16]. BoHV-1 was isolated from blood and nasal and vaginal secretions from each of the three vaccine groups. The heifers were ovariectomized nine days after vaccination for histological investigation and virus isolation. In all three groups necrotic oophoritis with multifocal areas of ovarian tissue necrosis, haemorrhage and mononuclear lymphocytic infiltration was observed. While some differences were observed between vaccine strains these were not statistically significant. Similar outcomes have been shown in pigs vaccinated with a pseudorabies (Aujezsky’s disease) virus vaccine [17], suggesting that the ability to induce necrotic oophoritis when administered at oestrus is a characteristic of alphaherpesviruses. It should be noted that studies (e.g. [16]) that describe pathological outcomes associated with IBR vaccine strains are included to illustrate the potential pathogenicity of BoHV-1, rather than of vaccine viruses as a generic group. Indeed the studies cited were typically conducted using early vaccines licenced for use outside Europe. The European Pharmacopoeia [18] that lays down the criteria for licensing of live IBR vaccines requires data from a minimum of 24 pregnant cows at various stages of gestation and administered ten times the vaccinal dose of virus to demonstrate the absence of either abortion or antibodies to BoHV-1 in pre-colostral sera.

A later study also investigated the impact of vaccine-induced changes on fertility [19], administering a modified live IBR vaccine i.m. to ten heifers that received two doses of prostaglandin ten days apart. Vaccine was given on the second of these treatment days at which time each animal in the trial was placed with a proven sire for 35 days and resulting conception rates were monitored. The conception rate in the control group of nine animals was determined to be 78% to first service rising to 100% following second service. In contrast only 30–40% of the ten heifers in the vaccinated group conceived to first service rising to 70% after second service. The authors attribute the marked difference in conception rates to a profound negative influence of concurrently administered vaccine virus on fertility.

#### Experimental studies-early pregnancy

All of the previous studies focused on infection with BoHV-1 at the time of oestrus. To evaluate the outcome of infection at different developmental stages of the bovine CL and conceptus, pairs of heifers were inoculated i.v. with the Iowa strain 7, 14, 21 and 28 days post natural service with post mortem examination 13–15 days after inoculation [20]. In contrast to the severe oophoritis reported in earlier studies associated with infection at oestrus, heifers inoculated at 7 or 14 days post natural service had mild oophoritis with a few necrotic follicles in one or both ovaries. Those inoculated 21 or 28 days post breeding had no lesions in the corpus luteum but numerous necrotic follicles in their ovaries, with viral antigen observed in all lesions by immunohistochemistry. The uterus of one heifer inoculated 7 days post-breeding contained a degenerating conceptus from which BoHV-1 was isolated. Heifers inoculated 14 days post-breeding were found not to be pregnant, but there was evidence that the post-breeding oestrus cycle had been longer than normal suggesting that conception followed by early embryonic death had occurred. The uteri of heifers inoculated at 21 or 28 days post-breeding (and the other heifer inoculated on day 7) contained normal-appearing concepti. Thus the results indicate that the pathogenic effect of BoHV-1 on the CL depends on the stage of development, with the severity of lesions decreasing as the interval from breeding to exposure increased. Shortly after ovulation, intense neo-vascularization develops in the follicular theca interna, and the authors speculate that infection occurring at oestrus can result in a large number of cells being simultaneously exposed to virus and leading to diffuse necrosis of the CL. In contrast, infection when the CL is fully functional and less vascularized may result in a lower level of exposure and subsequent pathology.

Having previously identified the diffuse necrosis of the CL and subsequent progesterone deficiency that accompanies infection at oestrus as one means by which BoHV-1 can prevent continued pregnancy, these authors considered cytocidal infection of the developing conceptus occurring when heifers are inoculated 7–14 days after breeding as a further mechanism by which infection may impact fertility. In this case embryonic death is not the result of luteal damage but rather cytocidal infection of the trophoblast.

In a subsequent study on the effect of BoHV-1 on fertility of heifers in the early embryonic period, the same researchers exposed 2 groups of recently served, seronegative heifers to BoHV-1 by the i.v. route either 7 or 14 days post-breeding [21]. Pregnancy occurred in all 5 control non-inoculated heifers, whereas only 1 of 8 inoculated heifers maintained pregnancy. These results were attributed to early embryonic death rather than a failure to conceive with inter-oestrus periods typically normal or only slightly longer than would be expected in the absence of conception. 3–4 months later all inoculated heifers were treated with dexamethasone and tissues subjected to histological and virological examination. Virus was isolated from the adrenal glands of 7 out of 8 of the challenged heifers and from vaginal and nasal swabs of 5 and 3 of these heifers respectively. Virus was also isolated from the reproductive tissues of one (ovary, infundibulum and uterine tube). Histological changes were observed only in the adrenal glands being characterised by a lymphohistiocytic infiltration accompanied less frequently by necrotic foci.

In an earlier study, the same researchers had shown that isolate FI had reduced pathogenicity compared to Iowa and Colorado strains in terms of its ability to cause endometritis and oophoritis when challenge occurred at the time of oestrous [13]. To further characterise this isolate they conducted a study to test its effect on pregnancy and determine whether it was pathogenic for the developing corpus luteum in heifers [8]. Nine seronegative heifers were bred naturally and inoculated i.v. with isolate FI (BoHV-1.2a) with 2 each being inoculated at 1, 7 and 14 days post-breeding (DPB) and the remaining 3 at 6 months gestation. Plasma progesterone was monitored to follow CL function with low values and failure to conceive being recorded for the 2 heifers inoculated at DPB 1. Luteal function was normal in heifers inoculated on DPB 7 and 14 with these 4 heifers producing healthy uninfected calves at term. These effects at DPB 7 and 14 differed from those observed with Iowa and Colorado strains (both BoHV-1.1 [20]). The 3 heifers inoculated with FI at 6 months all produced a calf at term although one was delivered dead. No virus was isolated from this calf but it was cultured from the placenta. The authors considered that BoHV-1 could be present in cotyledonary tissue without inducing lesions or infecting the foetus. The authors speculate that the FI isolate is less prone to cause reproductive failure because it is an IPV-type (BoHV-1.2) virus rather than an IBR (BoHV-1.1) type, with the former being considered as non-abortifacient. This was based on the observation that abortion is typically not seen in association with venereal disease (which is typically attributed to BoHV-1.2 strains) and that the recognition of the abortifacient activity of BoHV-1 coincided with its emergence as a cause of IBR in North America (with the latter typically attributed to BoHV-1.1 strains).

#### Epidemiological studies

Several epidemiological studies have investigated the impact of BoHV-1 on reproductive performance in dairy and beef herds. One of these [22] examined the impact of natural subclinical infections on fertility losses in a limited number of non-vaccinated dairy cows and heifers in Turkey. The study comprised 201 cows and 89 heifers cattle in 107 different herds, all of which were inseminated by a single individual using semen from the same bull. The average days open for cows that were seropositive at the time of service (99.3 ±16 days) was significantly higher than that recorded for seronegative cows (82.0 ±3.8 days). Conception rates were higher in seronegative (38.98%) than in seropositive cows (33.33%). Conversely, the conception rate was higher in seropositive (84.61%) than seronegative (56.57%) heifers, although in neither case were the differences statistically significant. The authors suggest that the better reproductive performance of seropositive heifers could be a consequence of their prior immunity to BoHV-1 at the time of service, although it might be expected that a similar protective effect would have been evident in the cows.

In a larger study of Estonian dairy herds comprising 9,637 animals in 65 seropositive and 38 seronegative herds, BoHV-1 was found to be significantly related to reproductive performance with the highest risk of increased insemination index and abortions occurring in herds with a moderate seroprevalence [23]. The odds ratios for these two events were 5.2 (95% C.I. 1.5–18.4) and 7.3 (95% C.I. 2.0–26.9) respectively. This study did not find a significant association between BoHV-1 and acute respiratory disease in adult cattle, although a separate analysis of the data did find a high occurrence of respiratory disease in unweaned calves to be associated with both a low to moderate (OR 14.8) and a high (OR 19.2) seroprevalence of BoHV-1 among cows [24].

A recent study of eight commercial dairy herds in Ireland [25] found a significant association between serological evidence of exposure to BoHV-1 and reduced conception rate.

In contrast a large study of Canadian beef herds [26] found no evidence of association between BoHV-1 serological status and reproductive performance.

### Abortion

The ability of the virus causing IBR to also induce abortions has been known for many years [27]. In North America the ability of both wild type field strains and early modified live vaccines to induce abortion has been recognised with one of the earliest reports from 1964 [28] describing the pathology of abortions due to BoHV-1 from a naturally occurring outbreak, experimental studies and following use of modified live vaccine in pregnant cattle. This study suggested an age-related susceptibility of the foetus with almost all abortions occurring in cows pregnant for at least six months. In the natural outbreak described in the same report there had been no obvious intercurrent disease seen in the herd. Similar lesions were found in all foetuses examined and distinctions could not be made between abortions caused by natural infection, experimental infection or vaccination. Characteristic gross lesions were not observed in any foetuses but on histological examination the typical lesion observed was of focal necrosis and this was considered to have considerable specificity for the disease. While focal necrosis was found in many tissues, it was best observed and occurred with greatest consistency in the liver. Lesions were also common in the spleen, thymic lobules, lymph nodes, kidney, adrenal cortex uterine endometrium and placenta.

A later epidemiological study [29] of 1,816 cows in 26 herds with abortion problems found a causal relationship between use of an early modified live IBR vaccine and abortion and infertility. The authors reported that cows vaccinated as adults with modified live vaccine prior to breeding had a 47.7% abortion rate, pregnant cows in contact with vaccinated calves had a 20.5% abortion rate and non-vaccinated exposed cows had an abortion rate of 17.9%.

A separate report [30] also described an outbreak of abortion in a dairy herd following i.m. administration of a modified live IBR vaccine. Of the thirty pregnant vaccinated cattle, twenty three pregnancies of four to six months duration were terminated by abortion or foetal death between 21 and 112 days after vaccination, representing a total pregnancy loss of 76.7%. Seven pregnancies yielded a mummified foetus.

In contrast, an early study [31] demonstrated the efficacy of an i.m. IBR vaccine against abortion. Challenges were conducted either i.n. or i.m. with one of two virulent field strains of BoHV-1: P8 isolated from respiratory disease in cattle and V11 isolated from an aborted foetus. Following challenge by either route, classical respiratory signs of IBR were seen in both control groups, albeit that a shorter and milder clinical course was observed following i.m. challenge, accompanied by a shorter duration of viral shedding from the nose. A high incidence of abortion occurred in control heifers with 10 of 16 heifers (62.5%) aborting compared to 1 of 17 (5.9%) of vaccinated heifers. Key observations in this study were that the incidence of abortion was not affected by factors including route of challenge inoculation, the strain of virus or the stage of pregnancy (3–6 months). Abortions in the control groups occurred between 8 and 41 days post challenge and the authors noted a greater success in isolating virus from placenta as compared with foetal tissues.

This observation was supported by the findings of other studies [32,33] which also highlighted placental tissue as a suitable diagnostic specimen, with the latter reporting the presence of culturable virus in the cotyledons of the placenta of 13 of 13 pregnant cows inoculated with BoHV-1 by the i.m. route. No virus-specific lesions were observed in the cotyledons of any of the 13 foetuses.

Other studies [34] have also demonstrated the efficacy of an intranasal IBR vaccine in preventing abortion. However, while the vaccine itself was shown to be both safe and efficacious in this study, the results from control groups challenged i.n. again demonstrated the abortifacient properties of BoHV-1. In the first control group foetuses averaged 8.5 months of age at challenge, ranging from 7.5–9 months. Out of 8 foetuses, 3 were born normally and remained healthy, 3 were born at term but died by 12 days of age, 1 was born prematurely, dying shortly after birth, and the final foetus was aborted. In the second control group foetuses averaged 6.5 months, (3.5–9 months) at the time of challenge. Only one offspring was considered normal with 2 being born alive but dying within one week and the remaining 5 being attributed to foetal wastage (abortions, mummification or premature birth). Thus, overall, 12 of 16 controls aborted, had a mummified foetus, foetus with latent infection or calved prematurely, or gave birth to calves that died by 12 days of age. The authors acknowledge that calves that died after birth may have contracted infection either *in utero*, by inhalation post-natally or by both routes.

A more detailed study [35] of 4 of these 5 calves that died shortly after birth reported lesions primarily characterised by focal necrosis distributed in many different tissues. In particular they highlighted the presence of these in the reticulum, rumen, omasum, abomasum, small and large intestines. Areas of haemorrhage were also present, particularly in the stomachs.

To further investigate the abortifacient properties of different BoHV-1 strains [10] 3 heifers were inoculated with the Cooper isolate (BoHV-1.1), 3 with the FI isolate (BoHV-1.2a) and 5 with the K22 isolate (BoHV-1.2b). Inoculation was carried out by the i.v. route 25–27 weeks after breeding. All heifers given the Cooper and FI isolates aborted 17–85 days after inoculation with foetal infection confirmed in all cases by immunohistochemistry and in some by virus isolation. All 5 heifers inoculated with K22 delivered full term calves. Placenta was available from 4 of these with K22 isolated from each one indicating that the virus had crossed the foetal–maternal barrier. In addition 4 of the 5 calves had BoHV-1 neutralising antibodies in pre-colostral sera indicating that K22 can infect bovine foetuses but may not be capable of killing them.

Overall the results suggested that BoHV-1.1 or 2a isolates caused abortion whereas BoHV-1.2b strains may infect the foetus but may be incapable of consistently causing foetal death. The authors noted the conflicting results in relation to the FI strain between this and their earlier study [8] and highlighted the need for repeated trials to characterise the abortifacient potential of individual strains. They also highlighted the problems associated with the use of BoHV-1 strains in respiratory vaccines available in the USA at that time due to their abortifacient potential in pregnant cattle.

However, while BoHV-1.1 strains are typically considered to be responsible for more severe clinical signs, they may also be associated with subclinical infections, including a report of a breakdown in a sero-negative 230 cow dairy herd in the United Kingdom [36]. Apart from slight watery ocular discharge in less than 5% of cows, no other clinical signs were observed.

A sub-clinical outbreak caused by BoHV-1.1 has also been described in a Dutch dairy herd [37]. Again, no clinical signs were observed in newly infected animals but detailed analysis of milk recording data showed a significant drop in milk production in animals that were initially seronegative. This production loss was estimated at approximately 9.5 litres per animal during the infectious period of 14 days. No pregnant cows aborted and overall there was no significant difference in the proportion of successful inseminations between cattle that were initially seropositive or seronegative.

In a study of 100 Estonian dairy herds [38], blood samples from 1,973 animals were tested, with herds being divided into 3 groups based on BoHV-1 seroprevalence (0, 1–49 and ≥50%). Herds in the 1–49% (OR 6.66, 1.82–24.35, 95% CI, p = 0.004) and ≥50% (OR 3.70, 1.00–13.71, 95% CI, p = 0.05) seroprevalence groups were found to have a higher odds ratio of having increased rates of abortion and herds in the highest seroprevalence group to have a trend toward a higher odds ratio for stillbirth (OR 3.38, 0.93–12.22, 95% CI, p = 0.06).

### History of IBR in the UK and Ireland

#### United Kingdom

IBR as a clinical entity was first described in the UK in 1961 [39] with the BoHV-1.2b (Oxford strain) isolated from this outbreak being designated as the British-type strain [6,7]. However it was not until the winter of 1977 that widespread reports of a sudden rise in the incidence and severity of IBR outbreaks, typically associated with high herd morbidity and variable but significant mortality, emerged in mainland UK.

This is illustrated by a review of changing trends in IBR in Great Britain [40] which analysed data from different sources covering the period from 1970–1986. Despite inherent limitations in the data, a significant increase in the incidence of IBR was evident in the late 1970s. This was associated with an increase in the prevalence of seropositive animals among healthy cattle, when it rose from less than 5% in the early 1970s to 10–12% in the mid 1980s, and in diseased cattle when it rose from 9.1% to 34.8% during the same period. Corresponding changes in herd seroprevalence occurred, increasing from 17.6% to 48%. A marked seasonal trend in respiratory disease attributed to IBR was apparent with a peak in the winter months. During this same period there was a significant rise in the number of BoHV-1-related abortions, which had a year-round distribution with a peak in July and August, consistent with autumn calving patterns.

A subsequent study [6] described the predominant genotypes of BoHV-1 in the UK. The majority of isolates analysed were made in England and Wales made between 1977 and 1987, but also included five isolates from Northern Ireland (N.I.) obtained between 1964 and 1984, the British type strain (Oxford) and four early isolates from the 1960s. All four of these early isolates were shown to be BoHV-1.2b strains. In contrast 66 of 73 isolates from Great Britain between 1977 and 1987 belonged to BoHV-1.1, with the remaining 7 belonging to BoHV-1.2b. There was no particular association between strain type and clinical signs, with ocular signs recorded for 5 of the 7 BoHV-1.2b isolates. All of the N.I. isolates were BoHV1.2b, with these frequently being found in association with respiratory disease. The authors hypothesised that a variant or mutant BoHV-1 emerged in the early 1950s in the USA and that on one or more occasion subsequently this new virus type was introduced to Europe through the importation of latently infected cattle.

To further investigate the differences in clinical response to different strains of BoHV-1, i.n. challenge studies were conducted in seronegative calves using BoHV-1.1 and BoHV-1.2b strains [7]. All calves developed clinically apparent disease post-challenge which was qualitatively similar for all six viral strains. However it was shown that quantitatively, clinical scores due to BoHV-1.1 strains were significantly higher overall than those due to BoHV-1.2b strains. Associated with this there was significant variation in cumulative virus shedding, being higher in BoHV-1.1 strains which also had significantly higher virus titres in nasal mucus. The authors consider this supporting evidence for the hypothesis that up to and including the 1960s, BoHV-1.2b strains were endemic in British cattle at low prevalence causing sporadic outbreaks of genital and relatively mild respiratory disease. From the mid 1970s onwards a more severe form of IBR associated with BoHV-1.1 emerged with a greater tendency to spread, reflecting higher titres of virus in nasal mucus and probably introduced with imported cattle from North America.

These findings are consistent with a much earlier publication [41] which reported a comparative i.n. study in calves with 3 European isolates of BoHV-1 from cases of IPV from Germany, Belgium and Austria and an isolate from a case of IBR in a Californian dairy herd. Calves inoculated with the Californian BoHV-1 isolate produced typical signs of IBR. Respiratory signs, although less marked, were also evident with the European isolates, with the milder response being attributed to a decreased affinity of the viruses for the respiratory tract tissues.

Consistent with the change in predominant strains of BoHV-1 in the UK, outbreaks of IPV are now rarely reported but do continue to happen. In one such case [42] an outbreak caused by a BoHV-1.2b strain occurred in a 147 cow dairy herd, being characterised by both genital and conjunctival signs. The infection was believed to have been introduced by a hired bull and presenting signs in the first cows affected included anorexia, milk drop, bilateral discharge with conjunctival inflammation and a vaginal discharge 3–5 days after service. These signs were accompanied by swelling of the vulva and excessive tail swishing. Cows were pyrexic with a mucopurulent vaginal discharge lasting 5–8 days and the vaginal mucosa was inflamed with numerous focal areas of ulceration, some of which coalesced, leading to the sloughing of a yellow- brown necrotic exudate. Respiratory signs such as coughing and nasal discharge were absent. Over the following 10–14 days 73% of the herd suffered milk drop, 86% had conjunctivitis and 81% had signs of genital infection. 46% of cows also had hyperaemic teats and discomfort milking, with all clinical signs being predominantly seen in younger cows. In an attempt to control the outbreak a live vaccine was given i.n. one week after the onset of clinical signs. This led to a rapid recovery of milk yield, although by this stage a loss of approximately 1000 litres had been recorded over a six day period. Despite revaccination 11 months later, 2 cows per week suffered milk drop during the subsequent milking season with teat discomfort again observed. A small number of infected cows had a mild genital infection characterised by a granular vulvitis which was considered indicative of a recurrence of latent infection. Infertility and abortion were not features of the outbreak. The same study [4] also reports other outbreaks where milk drop was recorded. In one case a 30% drop taking 3 months to recover was reported and in another individual losses were of the order of 15%.

In contrast to the situation in mainland UK the severe form of respiratory disease due to BoHV-1 was first diagnosed in N.I. in the early 1990s (author, personal observation).

#### Ireland

Historical information on BoHV-1 in the Republic of Ireland has been summarized previously [43]. The first isolate of BoHV-1 was made in 1971 from a case of conjunctivitis, with eleven more isolates made in the following decade, including one from a case of IPV, with no reports of severe IBR at that time. It was only in the latter half of the 1980s that the number of reported IBR outbreaks began to rise, with 102 isolates of BoHV-1 being made between September 1993 and March 1994.

The first published cases of severe respiratory disease associated with BoHV-1 in Ireland occurred in the winter of 1989/1990 in large feedlots in the mid-Leinster region and were characterised by high morbidity and high mortality, with infection diagnosed by immunofluorescence, virus isolation and rising antibody titres [44].

A later study [43] reported the clinical and molecular analysis of thirteen BoHV-1 isolates associated with outbreaks of disease in the Republic of Ireland between 1971 and 1992. Isolates from five outbreaks were identified as BoHV-1.2 viruses and the remaining eight to BoHV-1.1, IBR-like strains, the earliest of which was isolated in 1989. Overall, BoHV-1.1 strains were associated with more severe clinical signs, including systemic infection of neonatal calves.

Historically the seroprevalence to BoHV-1 in cattle in both N.I. and the Republic of Ireland was reported to be low. In the former, a seroprevalence of 13% was found in cattle older than nine months of age [45], while a 9% seroprevalence was found in cattle entering feedlots in the Republic of Ireland during the 1981/1982 season [44]. More recent studies have consistently reported markedly increased seroprevalence in both dairy and beef herds in Ireland. An investigation into the inter- and intra-herd seroprevalence of BoHV-1 in beef herds supplying bulls to an Irish performance testing station found herd prevalence to be 73.2% with a mean within herd prevalence of 28% (±20%) [46].

A study of 319 dairy herds over the course of their 2009 lactation found approximately 80% to have serological evidence of exposure to BoHV-1 based on multiple bulk tank tests with the majority of herds assigned a high positive score [47]. A serological survey of 1,175 Irish dairy and beef herds in 2009 using serum pools to estimate herd seroprevalence of 74.9% (95% C.I. 69.9%–79.8%) [48]. While neither of these studies was able to differentiate between infected and vaccinated herds, the finding that less than 2% of herds were vaccinating at the time supports the conclusion that infection is widespread in Ireland [48].

A similar marked rise in seroprevalence was reported in the UK [49], based on a longitudinal survey of 107 unvaccinated dairy and suckler herds in south-west England between 2002 and 2004. Overall 83.2% (95% C.I. 77.6%–88.8%) were found to contain one or more seropositive animal with 42.5% (95% C.I. 41.7%–43.3%) of animals in these herds always testing positive on serial sampling.

While no formal study of the association of BoHV-1 with abortion has been carried out in Ireland, surveillance data from the Veterinary Laboratory Service has found the virus in approximately 3% of foetuses by PCR (Dr. Ronan O’Neill, personal communication). This is consistent with results of recent reports from the UK and USA [50,51].

#### Wider European and US context

The detection of neutralising antibodies to BoHV-1 in sera from dairy herds from New York and New Jersey [41], almost 10 years prior to the recognition in 1954 of IBR as a disease entity in the USA [4]. This publication [41] also reports on the isolation of BoHV-1 from cattle from New York suffering IPV and presented a hypothesis for the emergence of clinical IBR, proposing that BoHV-1 was maintained in European cattle in the early 1900s primarily as a genital infection (IPV). This was favoured by the presence of small, relatively isolated herds with a predominance of natural service in Europe at that time, with little opportunity for the virus to undergo extensive serial propagation in the respiratory tracts of susceptible cattle. It was further proposed that the virus was introduced to the USA from Europe prior to 1930 when an embargo was imposed on the importation of cattle from continental Europe. Thereafter the virus initially became established in the eastern part of the US where the cattle population was comprised of mainly small isolated herds with husbandry practices similar to those in Europe that favoured venereal spread and limited opportunity for serial respiratory spread. When the virus gained entry to Western feedlots containing large numbers of susceptible animals, there was little opportunity for virus to be transmitted by the venereal route. However conditions were ideal for rapid serial passage of virus via the upper respiratory tract resulting in the selection of a virus population with a greater predilection for respiratory rather than reproductive epithelium. Consistent with this theory is the concurrent emergence of clinical disease in Californian dairy herds where cattle are maintained in conditions similar to those of large feedlots and AI rather than natural service predominated.

## Conclusions

IBR as a clinical entity first appeared in feedlots and dairies in the western USA in the 1950s. It is hypothesized that this reflected increased opportunity for, and an adaption to, replication in respiratory mucosa, leading to the emergence of BoHV-1.1 strains which evolved from IPV-like strains introduced previously from Europe. These more virulent IBR-like strains were in turn transferred to Europe, where severe respiratory disease emerged from the 1970s onward, becoming evident as a clinical problem in the UK in the 1970s and on the island of Ireland in the late 1980s/early 1990s. While IBR dominates the thinking of farmers and veterinary practitioners in relation to the clinical impact of BoHV-1 infection, there is a large body of evidence pointing toward negative reproductive outcomes that should not be overlooked or dismissed. While interpretation of some of the work is hindered by a lack of information on strain types and non-natural routes of infection, a number of points are evident. Firstly, infection at the time of service may reduce fertility, with the potential to cause chronic necrotising endometritis and oophoritis, accompanied by a shortened oestrous cycle. Infection later in the oestrous cycle may result in a decreased conception rate, whereas infection later in pregnancy can lead to abortion, mummification, stillbirth and the birth of live calves which die shortly thereafter. While negative reproductive outcomes are to some extent strain-associated (BoHV-1.1), such distinctions are not absolute. In an Irish context, the significance of these negative reproductive outcomes is unknown. Data from elsewhere [52] indicates that, even in endemically infected dairy herds many heifers are seronegative at calving, with the main reservoir of infection residing in the adult milking herd. Under such conditions it is not unreasonable to assume that, in the absence of vaccinal protection, such heifers may be exposed to BoHV-1 from carrier cows, with the outcome dependent, at least in part, on the stage of the oestrous cycle or pregnancy, the subtype of virus involved and the virulence of the particular strain. Studies to determine the epidemiology of infection in Irish dairy and suckler herds, and the prevalence of BoHV-1 subtypes therein are required. While challenging to conduct, such studies will be necessary to gain a better understanding of the dynamics of infection and the impact of, and losses associated with, BoHV-1 on reproduction.

## Abbreviations

AHI: Animal Health Ireland; AHWNI: Animal Health and Welfare NI; BoHV-1: Bovine herpesvirus 1; CL: Corpus luteum; DPB: Days post breeding; IBR: Infectious bovine rhinotracheitis; i.m.: Intramuscular; i.n.: Intranasal; IPB: Infectious vulvovaginitis; IPV: Infectious pustular balanoposthitis; i.v.: Intravenous; NI: Northern Ireland; UK: United Kingdom.

## Competing interests

The author declares that he has no competing interests.
